# A neural model of retrospective attention in visual working memory

**DOI:** 10.1016/j.cogpsych.2017.12.001

**Published:** 2018-02

**Authors:** Paul M. Bays, Robert Taylor

**Affiliations:** University of Cambridge, Department of Psychology, Cambridge CB2 3EB, UK

**Keywords:** Working memory, Retrospective cueing, Attention, Neural coding, Resource model

## Abstract

An informative cue that directs attention to one of several items in working memory improves subsequent recall of that item. Here we examine the mechanism of this retro-cue effect using a model of short-term memory based on neural population coding. Our model describes recalled feature values as the output of an optimal decoding of spikes generated by a tuned population of neurons. This neural model provides a better account of human recall data than an influential model that assumes errors can be described as a mixture of normally distributed noise and random guesses. The retro-cue benefit is revealed to be consistent with a higher firing rate of the population encoding the cued versus uncued items, with no difference in tuning specificity. Additionally, a retro-cued item is less likely to be swapped with another item in memory, an effect that can also be explained by greater activity of the underlying population. These results provide a parsimonious account of the effects of retrospective attention on recall and demonstrate a principled method for investigating neural representations with behavioral tasks.

## Highlights

• We fit a neural model of working memory storage to performance on retro-cue tasks.
• This model provided a better description of data than a prominent mixture model.
• Retro-cueing was associated with a higher firing rate of the encoding population.
• Results are consistent with protection of the cued item against temporal decay/drift.

## 1 Introduction

Recall of sensory information from short-term memory storage is imperfect; however, not all memory representations are alike: cueing paradigms have revealed that memory resources can be allocated under voluntary control. Surprisingly, benefits are observed not only for items cued at the time of presentation (Gorgoraptis et al., 2011, Sperling, 1960), but also for items cued retrospectively (Griffin and Nobre, 2003, Landman et al., 2003, Pertzov et al., 2013, Souza et al., 2014). In such retro-cueing experiments, one item from a visual memory array is indicated during the retention interval by, e.g., a spatial cue. This cue picks out an item that is more likely to be probed in the subsequent test display. Even though the items are no longer visible at the time of the cue, significant advantages in recall are observed for retro-cued items over other items in the array.

The mechanism by which retro-cue benefits arise has not yet been clearly delineated. Proposals include the removal from memory of redundant information related to uncued items (Souza & Oberauer, 2016), a strengthening of the cued item’s memory representation over and above its original encoding strength (Rerko et al., 2014, Souza et al., 2014, Souza et al., 2015), protection of the cued item from temporal decay or interference from other items (Pertzov et al., 2013, Pertzov et al., 2016), and protection from interference that arises from subsequent visual input (Makovski et al., 2010, Souza and Oberauer, 2016, Souza et al., 2016).

In recent retro-cue studies, the fidelity of recall has been investigated using the method of reproduction (e.g. Wilken & Ma, 2004) whereby participants report a probed feature from a memory array using an analogue (frequently circular) scale, such as a color wheel. Variability in feature reports produces a distribution around the true target value which typically differs from the circular normal (von Mises) distribution, having sharper peaks and longer tails. It has become common to analyse these responses using mixture models that describe errors as coming from one of several source distributions. In particular, response errors are often fit with a mixture of a normal distribution centered on the correct (target) feature value and a uniform distribution corresponding to random guesses (normal + uniform model; Zhang & Luck, 2008). A better fit is usually obtained if a further proportion of responses are drawn from normal distributions centered on non-target (unprobed) feature values, capturing “swap” errors (Bays et al., 2009, van den Berg et al., 2014).

Crucially, results from mixture modelling analyses have proven largely uninformative about the retro-cue benefit, with the majority of studies that have looked for them finding changes in all three mixture components: an increase in precision, a decrease in random responses, and a decrease in swap errors (Gunseli et al., 2015, Makovski and Pertzov, 2015, Murray et al., 2013, Souza et al., 2014, Souza et al., 2016, van Moorselaar et al., 2015, Wallis et al., 2015, Williams et al., 2013). The meaningfulness of model parameters is critically dependent on the correctness of the model, however previous retro-cue studies typically have not considered alternatives to the normal + uniform model.

Here, we consider a different perspective on visual working memory based on the principles of neural population coding (Bays, 2014, Bays, 2015, Schneegans and Bays, 2017). The population coding model accounts for recall errors by encoding stimulus features in the activation of a population of tuned neurons. Because neural firing is stochastic, decoding of the population rarely recovers stimulus information veridically, leading to errors of varying magnitude. Bays (2014) showed that this simple model accurately predicts the non-normality of error distributions observed in continuous report tasks. Additionally, by incorporating normalization of the population activity into the model, one can predict how error distributions will change with set size (the number of items in the memory array). This neural resource model provides a better fit to empirical data than the slot + averaging model of Zhang and Luck (2008), which extended the normal + uniform model to multiple set sizes.

Population coding is thought to be a fundamental mechanism of sensory representation found for different feature dimensions throughout cortex (Pouget et al., 2000, Zemel et al., 1998), and is therefore a strong candidate mechanism for storing working memories. For the sake of example, we will consider sensory responses of a typical orientation-selective neuron in primary visual cortex (Hubel & Wiesel, 1962). An oriented stimulus falling within this neuron’s retinotopic receptive field can elicit varying degrees of spiking. The primary factor driving cellular activity is where the orientation of the stimulus falls relative to the preferred orientation of the neuron. Feature values that coincide with the preferred value evoke a much larger response than do more distal feature values. The neuron’s tuning curve, then, is a function that describes how its activity changes with distance from the preferred orientation. Electrophysiological observations indicate that tuning curves are typically well-described by a bell-shaped function, scaled by a peak firing rate reflecting the response to the preferred stimulus value. The extent to which distal features evoke a response in any given neuron is determined by the tuning curve width, quantified as the full-width at half-maximum (FWHM). Narrow curves localize activity to features that are very close to the preferred value; broader curves spread activity across a wider range of features.

When fitting the neural model to data both the peak firing rate and tuning width are treated as free parameters; to simplify modeling, it is assumed that all neurons share these parameters, varying only in their preferred stimulus value (although it has been shown that the model predictions are not strongly dependent on this assumption; Bays, 2014).

Here we examine which parameters of the population coding model are affected by the retro-cue. In order to do so, we collated data from numerous previous studies so that we could maximize statistical power for the purpose of model fitting, and so as to ensure the generality of our results. We find that the tuning characteristics of the population coding model are uniquely affected by the retro-cue. Specifically, behavioral performance is consistent with higher overall firing rate of the population encoding the cued stimulus, while the tuning curve width is unaffected.

## 2 Method

### 2.1 Task and data

**Fig. 1:**
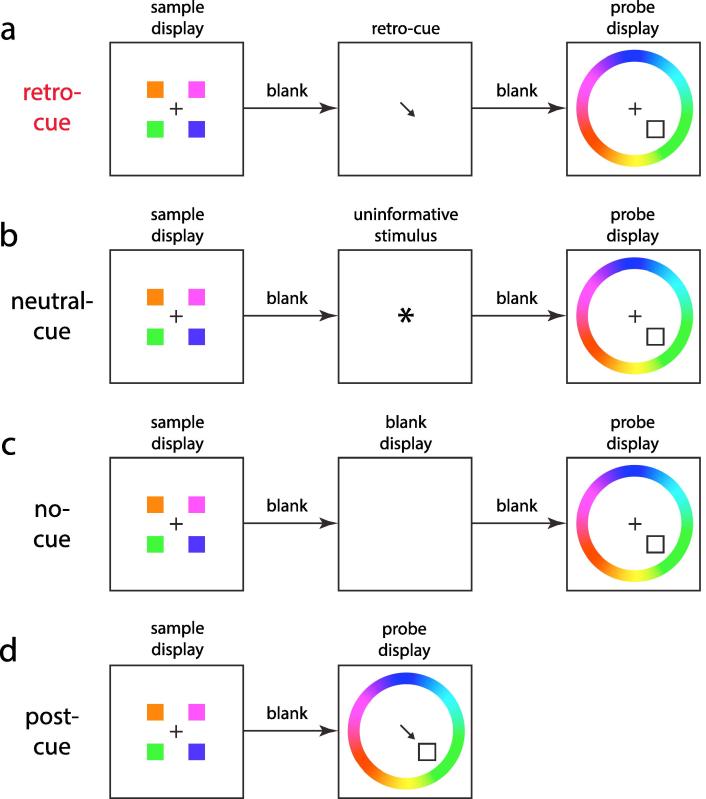
Illustration of experimental conditions. (a) Example of a retro-cue trial. A sample display containing one or more memory items (here, colored squares) is presented. After a blank delay period, a cue (here, an arrow) indicates one of the items in memory which is more likely to be probed. After a further blank delay, a probe (here, an empty square) is presented indicating one item that should be reported: in this example, a color wheel is presented simultaneously with the probe and participants click on the wheel to indicate the remembered color. (b) A neutral-cue trial. This control condition is identical to a retro-cue trial except an uninformative stimulus (here, an asterisk) is presented in place of the retro-cue. (c) A no-cue trial. In this control condition, no stimulus is presented during the memory period, i.e. the display remains blank between the sample display and probe. (d) A post-cue trial. In this control condition, the probe display is presented simultaneously with the retro-cue, indicating the same item. (For interpretation of the references to color in this figure legend, the reader is referred to the web version of this article.)

This study focuses on results from delayed estimation (continuous report) tasks, in which observers are required to report or reproduce a remembered stimulus on an analogue scale. These tasks involve three stages: a sample display, consisting of an array of items to be remembered, is followed by a blank delay period, during which the items must be held in memory, which is succeeded by a probe display, indicating which one of the items in memory is to be reported and typically providing the means of reporting it. A retro-cue (Fig. 1a) is a stimulus appearing during the delay period that indicates one of the items in memory that is more likely than the others to be subsequently probed.

We sent out requests for data to authors of every published study, to our knowledge, that has combined a delayed estimation task with a retro-cue condition. From the replies we obtained data from eight experiments from five different laboratories, comprising 188 participants and 65,492 trials (see Table 1). Experiments differed in a number of aspects, including the feature dimension reported, the number of memory items, the presentation timings of the memory items and cues, and the validity of the cue (the frequency with which it correctly indicated the item that would be probed). Only valid retro-cue trials were used for the analysis. As effects of set size were not the focus of the present study, where more than one set size was tested we treated each as an independent data set.

**Table 1:** Experimental studies.

No	Study	Report	Cue Validity	Control Cue	Set sizes	Participants	Trials
1	Williams et al. (2013), Exp 1	Color	100%	No-cue	2	20	200
2	Pertzov et al. (2013)	Orientation	70%	No-cue	4	24	350
3	Souza et al. (2014), Exp 1	Color	100%	Post	6	16	500
4	Souza et al. (2014), Exp 2	Color	100%	Post	2–8	21	868
5	Makovski and Pertzov (2015)^a^	Direction	100%	Neutral	3	42	160
6	Souza et al. (2016), Exp 3	Color	100%	Post	6	20	400
7	Souza (2016)^b^	Color	100%	No-cue	3, 5	24	100
8	Oberauer and Lin (2017), Exp 3	Color	66%	Neutral	2, 4, 6, 8	21	464

a Interference-absent condition.

b Young age group.

In addition to the retro-cue condition, one condition in each experiment was assigned as the control condition for purposes of our analysis. Three different types of condition could be designated as the control, to account for differences in methodology across studies. In neutral-cue conditions (Fig. 1b), a non-specific stimulus (e.g. an asterisk) that did not identify any individual memory item was presented in place of the retro-cue. In no-cue conditions (Fig. 1c), a blank screen was presented instead of the retro-cue. In post-cue conditions (Fig. 1d), the probe display was presented at the same time as the retro-cue, so the retro-cue provided no additional information beyond that provided by the probe itself. Where more than one of these conditions was present in an experiment we selected one to act as the control, taking in decreasing order of preference: neutral-cue, no-cue, post-cue.

**Fig. 2:**
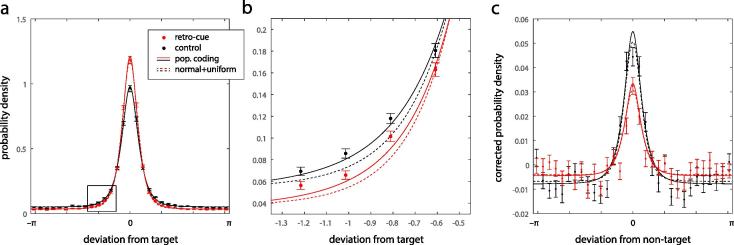
Error distributions. (a) Symbols indicate mean distribution of error around the target feature value in retro-cue (red) and control (black) conditions (errorbars indicate ±1 SE). Solid lines plot mean fits of the population coding model (incorporating swaps) for each condition; dashed lines show fits of the normal + uniform model (including swaps). (b) Close-up of the area marked by the rectangle in (a), revealing that the population coding model provides a closer approximation to the data than the normal + uniform model. Note that, because of the very large data set, this relatively small difference in fit provides overwhelming support for the population coding model. (c) Deviations of responses around non-target feature values: data and model fits as in (a). Probability density is corrected for chance (see Methods) such that responses would be uniformly distributed if non-targets had no influence on reports. (For interpretation of the references to color in this figure legend, the reader is referred to the web version of this article.)

Errors were calculated as the angular deviation between the feature reported by a participant and the true feature in the circular parameter space of possible feature values. We also calculated deviations of responses from non-target (i.e. unprobed item) feature values. Because a number of the experiments enforced minimum separations between stimuli in feature space, distributions of deviations from non-target values were corrected for chance using a randomization method: for each subject and condition, deviations of non-target feature values from target feature values were randomly shuffled, then the shuffled deviations were added back to the target feature values to generate new (simulated) non-target values; deviations of responses from these new non-targets were recorded. Averaged over 1000 repetitions, the distribution of response deviations provided an estimate of the chance distribution. This was subtracted from observed response frequencies to produce the corrected-for-chance histograms in Fig. 2c.

### 2.2 Models

Full mathematical details of the models are given in the Appendix. In the population coding model, each memory stimulus is encoded in the activity of a population of idealized neurons with von Mises (circular normal) tuning curves, and a recall estimate is obtained by optimal (*maximum a posteriori*) decoding of the population’s activity over a fixed time window. We described the neuronal tuning functions in terms of their height (the peak firing rate, $r_{\max}$), and width (the full-width at half-maximum, FWHM). These two free parameters fully determined the distribution of errors (deviations from the target stimulus value) predicted under the model. Code to fit the population coding model is available online at www.bayslab.com/code/JN14.

In the normal + uniform model, each memory stimulus is either stored (with von Mises distributed error) or not stored (resulting in random, i.e. uniformly distributed, error). The model has two free parameters: the probability of random response ($p_{\mathrm{guess}}$) and the concentration (inverse width) of the von Mises distribution ($\kappa^{\prime }$).

Both models can be extended by the addition of swap errors, i.e. responses incorrectly distributed around the non-target stimulus values. This results in an additional free parameter ($p_{\mathrm{swap}}$) determining the probability of a swap. We considered a further extension of the population coding model incorporating a probability ($p_{\mathrm{guess}}$) of responding at random.

Models were fit using the Nelder-Mead simplex method (*fminsearch* in MATLAB) to maximise likelihood of the model given the data. Model comparison was based on the Akaike Information Criterion (AIC). Statistical significance of differences in AIC scores was evaluated with sign tests across participants. Fitted parameters of both models were found to deviate from normal in their distribution (based on the Lilliefors test), so we used robust summary statistics and non-parametric hypothesis tests. Standard errors of the median were obtained by bootstrapping (1000 repetitions). Correlations were assessed on median parameter values obtained for each experimental data set: these were approximately normally distributed so parametric statistics and tests were used; specifically, the Pearson correlation coefficient and ANCOVA with condition (retro-cue vs control) as a factor.

## 3 Results

We examined the distribution of errors in recalling a memory item indicated by a probe, when the to-be-recalled item was signalled during the delay by a retro-cue (Fig. 2a & b, red symbols; control results without a retro-cue are shown in black). Confirming previous analyses (Makovski and Pertzov, 2015, Oberauer and Lin, 2017, Pertzov et al., 2013, Souza et al., 2014, Souza et al., 2016, Souza, 2016, Williams et al., 2013), recall was significantly more accurate on trials with a retro-cue present (mean absolute error, 30.9° vs 40.1°; t(17) = 7.2, p < 0.001). The mean error distributions plotted in Fig. 2a display the sharp peaks and long tails characteristic of working memory recall.

A model of working memory based on population coding provided a close fit to the data. The model fits are shown as solid lines in Fig. 2. As previously (Bays, 2014), the best fit to the data was obtained using a version of the model that incorporated swap errors, i.e. a possibility of inadvertently reporting one of the non-target (unprobed) items in the memory array (ΔAIC  = 2443; AIC favored swap model for 70% of participants; sign test, p < 0.001). Symbols in Fig. 2c plot the corrected (see Methods) distribution of errors around non-target values. If non-targets exerted no effect on responses these distributions would be uniform. Instead they show a central tendency consistent with the presence of swap errors. This central tendency was accurately captured by the model (solid lines).

**Fig. 3:**
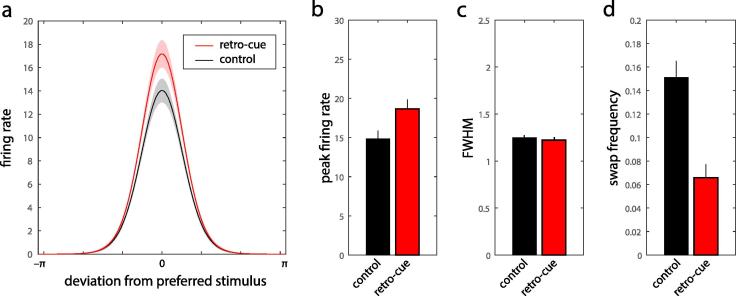
Estimated parameters of the population coding model. (a) Estimated tuning functions corresponding to performance in retro-cue (red) and control (black) conditions (median ±1 SE). Note the tuning function changes in gain (peak firing rate) between retro-cue and control conditions but there is no change in tuning width. (b–d) Median estimated parameters of the population coding model (errorbars indicate ±1 SE): (b) peak firing rate, (c) full-width at half-maximum (FWHM), (d) swap frequency. (For interpretation of the references to color in this figure legend, the reader is referred to the web version of this article.)

Fig. 3a shows the tuning functions estimated by the model to underlie representation of the memory stimuli, for retro-cue (red) and control (black) conditions. The height of the tuning function (i.e. the peak firing rate) was significantly greater in the retro-cue than control condition (Fig. 3b; $r_{\max}$, median 18.7 (retro-cue) vs 14.8 (control); Wilcoxon signed-rank test, p < 0.001). In contrast, the tuning width (full-width at half-maximum) did not differ between the two conditions (Fig. 3c; FWHM, 1.22 vs 1.25; p = 0.30). The estimated frequency of swap errors was significantly reduced in the retro-cue condition (Fig. 3d; $p_{\mathrm{swap}}$, 0.066 vs 0.15; p < 0.001).

**Fig. 4:**
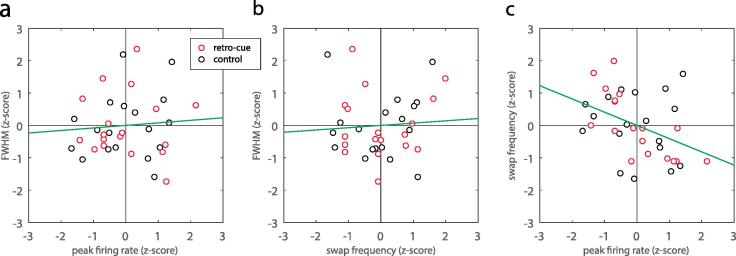
Correlations between parameters of the population coding model. (a) Peak firing rate vs full-width at half-maximum (FWHM). Each symbol indicates z-scored median parameter values for one experiment, in retro-cue (red) and control (black) conditions. Green line indicates best fitting regression line. (b) Swap frequency vs full-width at half-maximum (FWHM). (c) Peak firing rate vs swap frequency: note the strong negative correlation between these parameters. (For interpretation of the references to color in this figure legend, the reader is referred to the web version of this article.)

We observed no significant correlations between peak firing rate and tuning width parameters (r = 0.08, p = 0.86; Fig. 4a). However, we found a small but significant correlation between tuning width and swap frequency (r = 0.07, p = 0.048; Fig. 4b), and a strong, highly significant negative correlation between peak firing rate and swap frequency (r = −0.41, p = 0.003; Fig. 4c).

We compared the population coding model to an influential model of working memory in which memory items are either stored with circular normal (von Mises) distributed error, or not remembered at all, resulting in uniformly distributed error. Consistent with previous results (Bays et al., 2009, van den Berg et al., 2014) we again found that incorporating a probability of swap error into this model provided a superior fit to data (ΔAIC = 2600; 73% of participants; sign test, p < 0.001). Fits of the normal + uniform model with swaps are shown as dashed lines in Fig. 2. While both population coding and normal + uniform model predictions closely approximated the data, AIC scores indicated that the population coding model provided a better account overall (ΔAIC = 146; 63% of participants; sign test, p < 0.001; see Fig. 2b). ΔAIC scores greater than 10 are considered to indicate essentially no evidence for the weaker model (Burnham & Anderson, 2003).

Unlike the population coding model, all parameters of the normal + uniform model varied significantly between conditions: the retro-cue made the normal component more precise ($\kappa^{\prime }$, 12.0 (retro-cue) vs 10.8 (control); p < 0.001), and decreased the mixture proportions of uniform ($p_{\mathrm{guess}}$, 0.06 vs 0.12; p < 0.001) and swap components ($p_{\mathrm{swap}}$, 0.038 vs 0.091; p < 0.001).

The population coding model assumes that all items enter memory, and while recall errors may be arbitrarily large, there is no explicit guessing. To test the validity of this assumption, we modified the population coding model to incorporate a proportion of random responses. AIC scores indicated that this provided a less parsimonious account of the data than the unmodified model (ΔAIC = 188; 87% of participants; sign test, p < 0.001; $r_{\max}$, 26.1 (retrocue) vs 23.9 (control); FWHM, 1.48 vs 1.66; $p_{\mathrm{swap}}$, 0.048 vs 0.12; $p_{\mathrm{guess}}$, 0.017 vs 0.062).

## 4 Discussion

We tested the ability of a population coding model of working memory to account for the effects of a retrospective cue on recall. We found that behavioral data drawn from existing studies were consistent with a relatively enhanced firing rate of the population encoding a cued item, whereas tuning width remained unchanged between cued and uncued items. The frequency of swap errors, in which a different item in memory is reported than the one probed, was also substantially reduced for retro-cued items.

These observations parallel neurophysiological results related to prospective orienting of attention to visual stimuli, where it has been shown that directing attention to a cued stimulus scales the firing rate of sensory neurons without a change in tuning width (McAdams and Maunsell, 1999, Motter, 1993). This principle has been incorporated into neural models of attention which have been shown to accurately describe physiological and behavioral data (Reynolds & Heeger, 2009). The elevation in activity can be straightforwardly modeled as a multiplicative increase in the driving input to neurons encoding the cued item, and this principle has previously been successfully applied to model effects of prospective cues on working memory (Bays, 2014).

However, a critical difference from prospective cueing is that in retro-cueing it is not possible to extract additional information about the cued item from sensory input, as it has already been removed. This means that the retro-cue cannot increase the total information stored about a stimulus. A mechanism that boosted activity of a population corresponding to the retro-cue indiscriminately, i.e. without access to new information about the encoded stimulus, would amplify the noise as well as the signal, and – at least for an optimal decoder – not produce a benefit. This consideration suggests the possibility that the relative increase in firing rate reported here could in fact reflect a preservation of the activity encoding the cued item, while activity associated with the uncued items is diminished. This would be consistent with the proposal that retro-cueing protects items from time-based decay (Pertzov et al., 2013, Pertzov et al., 2016).

The same focus of attention that sustains activity may also protect cued items from perceptual interference, whereby presentation of subsequent visual information (e.g., visual masks, probe arrays) corrupts the representation of uncued items (Makovski et al., 2010, Souza et al., 2016). Retro-cuing benefits have been observed in control conditions that equate the retention interval with the time of retro-cue onset, suggesting that the retro-cue effect, at least in part, reflects protection from interference by the probe array. Speculatively, the effect of the new visual stimulation could be modeled as a random pattern of activations of the neural population: stimulus estimates obtained from a population with high gain will be distorted far less by additional random spikes than from a population with weaker levels of activity. This could be an interesting direction for future research, but instantiating such a model and fitting it to data lies outside the scope of the current study.

An alternative hypothesis to temporal decay is the possibility of drift in the encoded stimulus value during the retention interval. Maintaining a stimulus representation in persistent activity is thought to require a self-excitatory process whereby neurons continuously refresh their firing rate based on preceding activity (Burak and Fiete, 2012, Wimmer et al., 2014). Because spiking is probabilistic, the encoded value changes over time, taking a random walk from its initial value. Response precision is accordingly time-dependent, with longer retention intervals making representations less reliable. While distinguishing between drift and decay accounts poses an interesting question for future research, these hypotheses are not dissociable based on the present evidence.

In addition to drift, certain dynamic models of working memory maintenance (e.g. Wei, Wang, & Wang, 2012) propose that competition between representations can lead to the ”sudden death” of items in memory, with the frequency of such events increasing with the number of memoranda. However, direct neurophysiological evidence for such a mechanism is lacking, and because sudden death would manifest as a uniform component in response distributions, the present results indicate this mechanism is not needed to explain behavioral data either. On the other hand, the population coding model presented here merely assumes that representations can be maintained over time, without providing a neural mechanism for it, and this is an important direction for future development.

The tendency to report features of unprobed items (i.e. swap errors) is thought to arise from variability in the representation of the probe feature dimension, typically spatial location (Bays et al., 2009). In other words, swaps occur because sometimes the wrong item is remembered as closest to the probe location. Here we simply estimated swap frequency by centering decoding distributions on each of the non-target values and leaving their mixture proportion as a free parameter. However, recent work (Schneegans & Bays, 2017) has shown that swap error frequencies can be accurately predicted by a conjunction coding model in which neurons have spatial as well as feature tuning. The negative correlation observed here between swap frequency and peak firing rate is consistent with this model: enhanced activity would be expected to increase the precision with which spatial as well as feature information is maintained, reducing the probability of mislocalizing the target according to the conjunction model. The weak correlation observed between swap frequency and tuning width might reflect a positive relationship between tuning for the reported feature and space.

Once swaps were included, the widely-used normal + uniform mixture model provided an adequate description of error distributions, but we found—consistent with previous results—that no single parameter of the model was uniquely affected by the retro-cue. Precision of the normal component increased and mixture proportions for both the uniform and swap components decreased for cued items relative to controls. These results preclude drawing any firm conclusions about the mechanism of retro-cue benefits based on this model.

A critical assumption of the normal + uniform model, distinguishing it from the population coding model, is that features in memory are recalled with von Mises (circular normal) distributed error. If the true distribution deviates from this, estimates of the uniform component could be substantially incorrect. To our knowledge, no justification for the von Mises assumption has been put forward, but we presume it reflects the fact that the von Mises is often considered a circular analogue of the normal distribution in Euclidean space (hence the term circular normal). In fact, the von Mises does not possess many of the important properties of the Euclidean normal, but it does serve as an approximation to the wrapped normal distribution, which is a central limit distribution on the circle (Jammalamadaka & Sengupta, 2001). A central limit distribution is a distribution to which the sum or average of a set of samples converges as the number of samples becomes large. However, it is important to recognize that this convergence occurs much more rapidly in the body of the distribution than in the tails, and is only complete when the number of samples is infinite (Chen & Shao, 2001). The output of the population coding model shows that optimal decoding of finite numbers of spikes produces distributions that deviate significantly from the von Mises, particularly by virtue of their long tails. While swaps also contribute to the non-normality of observed errors, the present study shows that these distributions provide a better account of the data than the normal + uniform model.

Model comparison based on data from more than 65,000 trials found strong evidence supporting the population coding model over the normal + uniform model. Perhaps even more importantly, the population coding model describes a biologically-plausible process by which the observed errors arise. In contrast, the normal + uniform model merely categorizes errors into two classes, without explaining how the errors in each class are generated. The normal + uniform model is associated with the concept of a fixed capacity limit and specifically the slot + averaging model of Zhang and Luck (2008). However previous work has shown that this latter model fits the data poorly, firstly because—once swaps are taken into account—the estimated frequency of guessing is not consistent with a fixed upper limit (Bays et al., 2009), and secondly because it fails to account for the deviations from normality observed at lower set sizes, including recall of a single item (Bays, 2014, Bays, 2015). The population coding model, by contrast, belongs to the resource family and does not impose a limit on the number of items that can be stored in memory. While for some the need for a fixed working memory limit at three or four items remains a contentious issue, we believe the consistent superiority of models that do not incorporate such a limit in fitting empirical data cannot be ignored (Bays, 2014, Fougnie et al., 2012, van den Berg et al., 2012, van den Berg et al., 2014).

Does the success of the population coding model mean that there are no true guesses amongst working memory responses? We found that adding a random response distribution to the output of the population coding model produced a worse model as assessed by AIC, confirming that describing responses in terms of population coding eliminates the need for a separate guessing process. However, not all items will generate spiking patterns that permit unambiguous decoding of feature values, and the accuracy and confidence in decoded estimates will vary from item to item and trial to trial (cf. Fougnie et al., 2012, van den Berg et al., 2012). As a result, some very inaccurate, low confidence estimates will be generated, of the kind that could reasonably be described as “guesses”. Critically, rather than originating from a separate process, these responses are simply one end of a continuum of errors predicted by population coding, at the other end of which are highly veridical, high certainty estimates.

To conclude, we have shown how the population coding model provides a biologically plausible interpretation of the retro-cue effect. More generally, the population coding model offers both a substantive, and pragmatic, alternative to current models of visual working memory. Furthermore, it has a demonstrated ability to better approximate empirical data, making it an invaluable tool for investigating visual working memory performance.

## Data Availability

All data associated with this article can be found at https://osf.io/58q4r.

## Appendix A

### A.1 Population coding model

In the population coding model, a memory stimulus is encoded in the activity of a population of neurons with homogeneous tuning, and a recall estimate is obtained by optimal decoding of the population’s activity over a fixed time window. Mathematically, the model is described as follows.

Consider a population of *M* idealized neurons with von Mises tuning functions. The average response of the *i*th neuron is defined as:

(1) $$r_{i}(\theta )=\frac{\gamma }{M}\frac{1}{I_{0}(\kappa )}\exp(\kappa \cos(\theta -\varphi_{i}))\text{,}$$

where θ is the stimulus feature value, $\varphi_{i}$ is the neuron’s preferred feature value, γ is the population gain and κ is a scale parameter. $I_{0}(\cdot )$ is the modified Bessel function of the first kind with order zero. Preferred feature values were evenly distributed throughout the circular space of possible values.

Spiking activity was modeled as a homogeneous Poisson process, such that the probability of neuron *i* generating *n* spikes in time *T* was:

(2) $$\mathrm{Pr}\left[n_{i}\right]=\frac{{\left(r_{i}(\theta )T\right)}^{n_{i}}}{n_{i}!}\exp\left(-r_{i}(\theta )T\right)\text{.}$$

Recall of the stored feature was modeled as decoding of feature information from the population’s spiking activity, n, over a fixed time period, based on *maximum a posteriori* (MAP) decoding. Assuming a uniform prior, this is equivalent to maximizing the likelihood:

(3) θ^=arg maxθ′Prn|θ′.

If two or more feature values tied for the maximum, the decoded value was sampled at random from the tied values.

Eqs. (1), (2), (3) provide a full description of the model; further simplifications allow for an efficient method of obtaining predicted error distributions. Following Bays (2016), it can be shown that, for M→∞, the error in the decoded estimate Δθ=θ⊖θ^ is distributed as the resultant angle of a Von Mises random walk of *m* steps (⊖ indicates subtraction on the circle). It follows that the error for a given resultant length *r* is Von Mises distributed

(4) $$p(\Delta \theta |r\text{,}\kappa )=\frac{\exp(\kappa r\cos\Delta \theta )}{2\pi I_{0}(\kappa r)}\text{,}$$

where the distribution of *r* for *m* steps is given by

(5) $$p(r|m\text{,}\kappa )=\frac{I_{0}(\kappa r)}{{\left(I_{0}(\kappa )\right)}^{m}}r\psi_{m}(r)\text{,}$$

where $r\psi_{m}(r)$ is the probability density function for resultant length *r* of a uniform random walk of *m* steps. The distribution of *m*, the total spike count during the decoding interval *T*, being a sum of *M* independent Poisson distributions, is itself Poisson:

(6) $$\mathrm{Pr}[m]=\frac{\xi^{m}e^{-\xi }}{m!}\text{,}$$

where ξ=γT is the expected total spike count. For simplicity we set the decoding period to 1 s (changing this value would merely result in a corresponding change to the estimated gain parameter γ, e.g. setting *T* = 0.1 s would multiply the gain by 10).

The parameterization of the model in terms of κ and γ, while providing a mathematically compact description of the tuning function (Eq. 1), is not ideal for interpretation because the value of κ affects both the width and height of the tuning curve. To provide more directly interpretable parameters, therefore, we reparameterized the model in terms of the height (peak firing rate; $r_{\max}$) and width (full-width at half-maximum; FWHM), of the tuning function. To obtain $r_{\max}$ we note that Eq. 1 has a maximum at $\theta =\varphi_{i}$, implying:

(7) $$r_{\max}=\gamma \frac{1}{I_{0}(\kappa )}\exp(\kappa \cos(0))=\frac{\gamma e^{\kappa }}{I_{0}(\kappa )}\text{.}$$

where we have dropped the dependence on *M* as it would provide only an arbitrary scaling in this context.

To find the FWHM we first seek the half-maximum points $\theta_{\mathrm{HM}}$. For this purpose, the normalization constant may be safely ignored, so we focus only on the second term in Eq. 1. Noting again that $r_{i}(\theta )$ reaches its maximum when $\theta =\varphi_{i}$, we have:

(8) $$\begin{array}{c} e^{\kappa \cos(\theta_{\mathrm{HM}}-\varphi_{i})}=\frac{1}{2}e^{\kappa }\\ \kappa \cos(\theta_{\mathrm{HM}}-\varphi_{i})=\log(0.5)+\kappa \\ \cos(\theta_{\mathrm{HM}}-\varphi_{i})=1+\frac{\log(0.5)}{\kappa }\\ \theta_{\mathrm{HM}}=\varphi_{i}\pm \cos^{-1}\left(1+\frac{\log(0.5)}{\kappa }\right)\text{.} \end{array}$$

The resulting half-maximum points lie on either side of $\varphi_{i}$, so the FWHM corresponds to the distance between $\theta_{\mathrm{HM}+}$ and $\theta_{\mathrm{HM}-}$,

(9) $$\text{FWHM}=\theta_{\mathrm{HM}+}-\theta_{\mathrm{HM}-}=2\cos^{-1}\left(1+\frac{\log(0.5)}{\kappa }\right)\text{.}$$

We considered an extension of the population coding model that incorporated swap errors, i.e. a possibility of incorrectly reporting one of the unprobed items in memory. Defining as f(·) the probability distribution of errors around a stored feature value predicted by the population coding model above, the probability of response θ^ under the extended model is given by:

(10) p(θ^)=(1-pswap)f(θ^⊖θ)+1N-1pswap∑j=1N-1f(θ^⊖ψj),

where *N* is the number of items in the array, θ is the probed item feature, and $\psi_{j}$ is the *j*th unprobed item feature.

A further extension was considered that incorporated a probability of responding at random, $p_{\mathrm{guess}}$:

(11) p(θ^)=(1-pswap-pguess)f(θ^⊖θ)+1N-1pswap∑j=1N-1f(θ^⊖ψj)+pguess12π.

### A.2 Normal + uniform model

In the normal + uniform model, the distribution of responses θ^ comprises a mixture of von Mises (circular normal) and uniform components:

(12) p(θ^)=(1-pguess)VM(θ^;θ,κ′)+pguess12π,

where VM(x;μ,κ) is a von Mises distribution with mean μ and concentration κ, $p_{\mathrm{guess}}$ is the probability of a random response, $\kappa^{\prime }$ is the concentration of ‘remembered’ responses. The free parameter $\kappa^{\prime }$ is written with a prime symbol (${}^{\prime }$) to distinguish it from the variable used in the description of the population coding model.

We again considered an extension of the population coding model that incorporated swap errors,

(13) p(θ^)=(1-pswap-pguess)VM(θ^;θ,κ′)+1N-1pswap∑j=1N-1VM(θ^;ψj,κ′)+pguess12π,

where *N* is the number of items in the array, θ is the probed item feature, and $\psi_{j}$ is the *j*th unprobed item feature.
